# Visualization of HIV-1 Interactions with Penile and Foreskin Epithelia: Clues for Female-to-Male HIV Transmission

**DOI:** 10.1371/journal.ppat.1004729

**Published:** 2015-03-06

**Authors:** Minh H. Dinh, Meegan R. Anderson, Michael D. McRaven, Gianguido C. Cianci, Scott G. McCoombe, Z. L. Kelley, Casey J. Gioia, Angela J. Fought, Alfred W. Rademaker, Ronald S. Veazey, Thomas J. Hope

**Affiliations:** 1 Division of Infectious Diseases, Department of Medicine, Northwestern University Feinberg School of Medicine, Chicago, Illinois, United States of America; 2 Department of Cell & Molecular Biology, Northwestern University Feinberg School of Medicine, Chicago, Illinois, United States of America; 3 School of Medicine, Deakin University, Melbourne, Australia; 4 Department of Preventative Medicine, Biostatistical Collaboration Core Center, Northwestern University, Chicago, Illinois, United States of America; 5 Department of Comparative Pathology, Tulane National Primate Research Center, Covington, Louisiana, United States of America; Emory University, UNITED STATES

## Abstract

To gain insight into female-to-male HIV sexual transmission and how male circumcision protects against this mode of transmission, we visualized HIV-1 interactions with foreskin and penile tissues in *ex vivo* tissue culture and *in vivo* rhesus macaque models utilizing epifluorescent microscopy. 12 foreskin and 14 cadaveric penile specimens were cultured with R5-tropic photoactivatable (PA)-GFP HIV-1 for 4 or 24 hours. Tissue cryosections were immunofluorescently imaged for epithelial and immune cell markers. Images were analyzed for total virions, proportion of penetrators, depth of virion penetration, as well as immune cell counts and depths in the tissue. We visualized individual PA virions breaching penile epithelial surfaces in the explant and macaque model. Using kernel density estimated probabilities of localizing a virion or immune cell at certain tissue depths revealed that interactions between virions and cells were more likely to occur in the inner foreskin or glans penis (from local or cadaveric donors, respectively). Using statistical models to account for repeated measures and zero-inflated datasets, we found no difference in total virions visualized at 4 hours between inner and outer foreskins from local donors. At 24 hours, there were more virions in inner as compared to outer foreskin (0.0495 +/− 0.0154 and 0.0171 +/− 0.0038 virions/image, p = 0.001). In the cadaveric specimens, we observed more virions in inner foreskin (0.0507 +/− 0.0079 virions/image) than glans tissue (0.0167 +/− 0.0033 virions/image, p<0.001), but a greater proportion was seen penetrating uncircumcised glans tissue (0.0458 +/− 0.0188 vs. 0.0151 +/− 0.0100 virions/image, p = 0.099) and to significantly greater mean depths (29.162 +/− 3.908 vs. 12.466 +/− 2.985 μm). Our *in vivo* macaque model confirmed that virions can breach penile squamous epithelia in a living model. In summary, these results suggest that the inner foreskin and glans epithelia may be important sites for HIV transmission in uncircumcised men.

## Author Summary

Although several clinical trials have demonstrated that male circumcision can protect men from becoming infected with HIV, we know very little about how men get infected through sex and how circumcision changes this. In this study, we explored possible sites of virus transmission across the penis by looking at how HIV interacts with adult male foreskins, penile tissues from circumcised and uncircumcised cadavers, and male rhesus macaques. Using epifluorescent microscopy, we captured images of individual HIV particles entering the penile skin, sometimes to depths where CD4+ (potential target) cells could be found. We found more virus in and on the inner aspect of the foreskin than the outer aspect of the foreskin after culturing for 24 hours. Additionally, there was more virus entering the glans penis as compared to foreskin tissues from uncircumcised cadaveric donors, and to greater depths in these tissues. We made similar observations of virus entering the tissue in living rhesus macaques, strengthening the results obtained from human tissues. This information should help us better understand how the virus moves into uncircumcised penile tissue placing uncircumcised men at higher risk for HIV infection during sex.

## Introduction

The World Health Organization estimates that over 35 million people world-wide are currently infected with the human immunodeficiency virus (HIV)[1]. The majority of these infections are acquired through heterosexual transmission events, with female-to-male HIV transmission rates approaching that of male-to-female in some areas[2]. Male circumcision has been shown to effectively reduce the risk of HIV acquisition in men by 50–60% in three large African cohorts[3–5]. This protective effect appears to be long-lasting and extends to other sexually transmitted infections (STIs) such as human papillomavirus and herpes simplex virus-2[6]. In contrast, the benefits of male circumcision have not been so clearly defined for men who have sex with men[7,8]. Our lack of a scientific model for how HIV infects the man through the penis hinders our ability to explain how male circumcision protects against HIV infection, as well as to interpret these clinical disparities. In this study, we sought to explore potential sites of HIV transmission through the penis using tissue explants from adult donors and a living rhesus macaque model.

In all primates, the penis is naturally covered with a prepuce or foreskin. The foreskin is composed of an “inner” aspect that is adjacent to the glans epithelia in the flaccid state. The inner foreskin attaches to the penis at the coronal sulcus. In the erect state, the foreskin retracts to expose the inner surface to the environment. The “outer” foreskin is continuous with the penile shaft and remains exposed to the environment in both flaccid and erect states. An initial hypothesis for HIV entry into the uncircumcised penis centered on differences in foreskin keratin layers (or stratum corneum, SC)[9,10]. A thin inner foreskin SC would allow the virus to more easily penetrate the skin and encounter a HIV susceptible immune cell (e.g., Langerhans cells, CD4+ T-cell lymphocytes or macrophages). However, quantitative studies using foreskins from donors in China, the USA, and Uganda found no biologically or statistically significant difference in SC thickness between foreskin areas[11–13]. Other studies have demonstrated that the surface area of the foreskin correlates with HIV incidence rates, suggesting that simple removal of this target cell-rich tissue would be sufficient to lower a man’s risk of HIV sexual acquisition[14,15]. This risk may also be influenced by factors that have been shown to differ between circumcised and uncircumcised men, such as hygiene practices, latent STIs, and bacterial colonizers[16–18]. Latent STIs may also alter target cell populations in the tissue by recruiting cells to the surface or activating them, and thus enhance HIV susceptibility[19,20]. Finally, the increased HIV incidence rates in vaccinated, uncircumcised male subjects in the Merck HIV-1 STEP trial support the idea that the vaccine elicited a mucosal response and subsequently enhanced HIV transmission in the male genital tract[21]. These studies collectively raise questions on how penile tissues change after circumcision and how these changes contribute to HIV transmission through the penis.

In this report, we investigated how HIV-1 interacts with male penile stratified squamous epithelia by visualizing and characterizing the earliest interactions of photoactivatable GFP-labeled HIV-1 with human and rhesus macaque penile tissues. We also surveyed tissue-resident immune cells in these tissues and found potentially important differences between foreskin, glans, shaft, and urethral meatus tissues. This information will help guide future studies on how male circumcision affects HIV sexual transmission through the penis.

## Results

### HIV-1 Interactions with Foreskin Tissues Obtained from Local Donors

**Fig 1:**
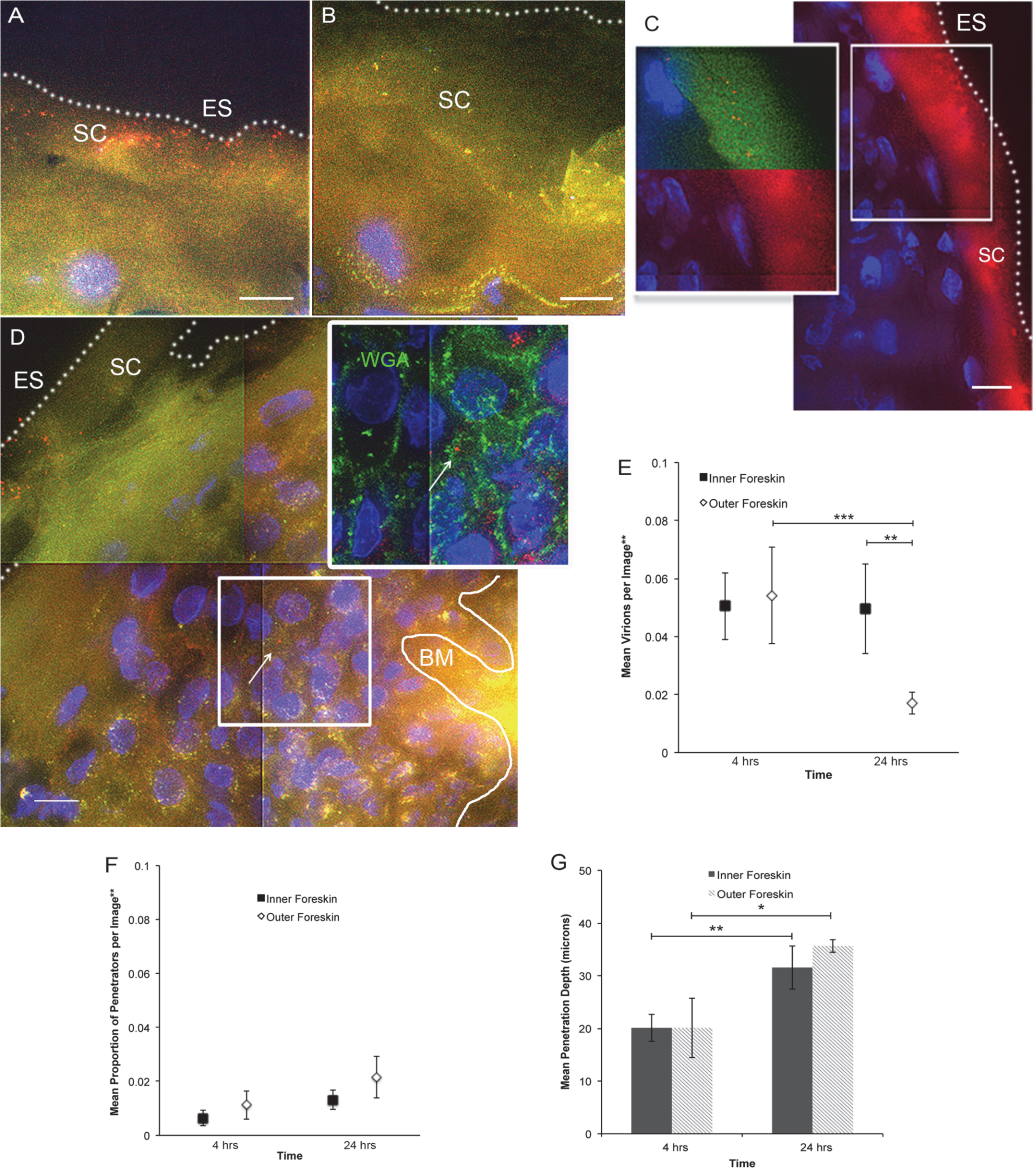
*Ex vivo* PA GFP HIV-1 interactions with adult human foreskin tissues. Foreskins obtained from consenting adult donors and inoculated with R5-tropic PA GFP-Vpr HIV-1 for 4 (n = 10) or 24 hours (n = 12) in culture. (A) and (B) Representative images of virion interactions with inner (A) and outer (B) foreskins after 4 hours of HIV exposure *ex vivo*. When seen, virions (red) were found predominantly on the surface or in the stratum corneum (SC). ES, dotted line, epithelial surface. (C) When co-inoculated with fluorescently labeled bovine serum albumin (BSA, red, right panel), virions (red, top half of inset, pseudo-colored to reveal PA GFP) were seen diffusing to depths that BSA also reached. (D) The majority of penetrating virions (virions seen below the SC) were found interstitially, as determined by tissues stained with fluorescent wheat germ agglutinin (WGA, green, inset). All images: white bar = 10 μm, blue = cell nuclei. (E-G) Estimated means of total virion counts (E), ** = adjusted for virus stock concentrations; proportion of penetrators (F); depths of penetration (G). Dark squares and bars represent inner foreskin; open diamonds and bars represent outer foreskin. *p<0.05, **p<0.01, ***p<0.001

Fluorescently labeled CCR5-tropic (R5-tropic) HIV-1 was made by co-transfecting 293T cells with an HIV-1 provirus and photoactivatable GFP-Vpr constructs (PA GFP HIV)[22–24]. Foreskin tissues were obtained from local consenting adult donors and cultured with PA GFP HIV for 4 and 24 hours (n = 10 and 12, respectively). 1612 images of tissue cryosections were obtained using deconvolution epifluorescent microscopy. A subtraction method was used to determine true PA GFP HIV from tissue background autofluorescence, as previously described[24]. Many images captured did not contain virions (∼40%); in those that did, we counted 15626 individual virions, the majority of which were found on the epithelial surface or in the stratum corneum (SC) (Fig. 1A and 1B). Foreskin specimens inoculated with PA GFP HIV and a fluorescent fluid phase marker (bovine serum albumin, BSA) demonstrated that the virus diffused into the SC in a similar manner as BSA (Fig. 1C). That is, there was heterogeneous distribution of both BSA and virions into the SC, with some areas allowing for shallower diffusion and other areas allowing for deeper diffusion. These patterns did not demonstrably differ between the inner and outer foreskin. On average, 1 per 100 virions visualized were seen past the SC, which we termed, “penetrators” (Fig. 1D). The range of penetration depths seen in foreskin tissue was 0–96.69 μm (S1A Fig.). Using wheat germ agglutinin to highlight epithelial cell surfaces, we determined that >80% of penetrators were found between rather than inside a cell (inset, Fig. 1D).

**Fig 2:**
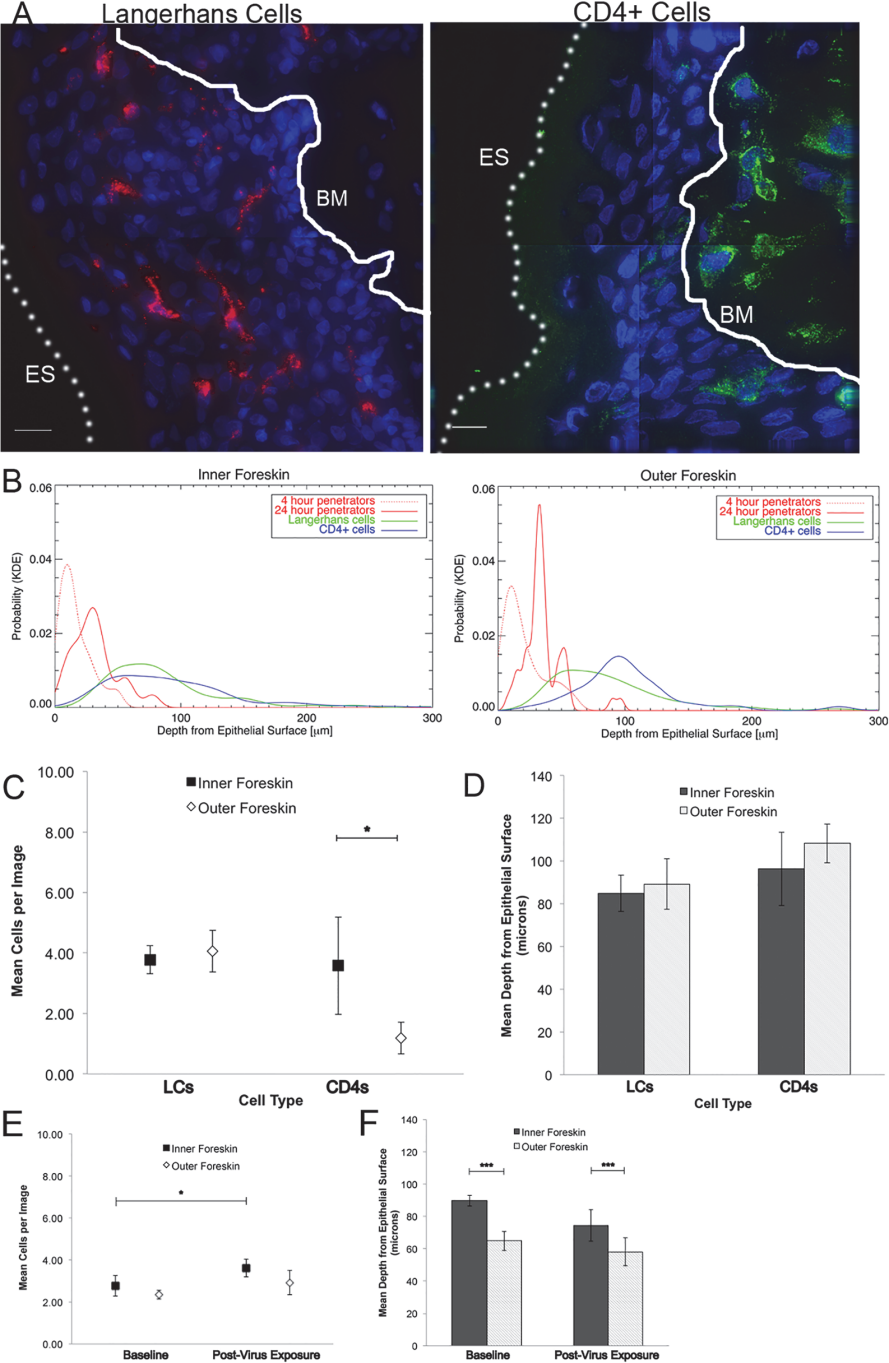
Tissue resident immune cells in foreskin tissues. Tissue cryosections immunofluorescently stained with OKT6 or α-CD4 antibodies to detect Langerhans cells (LCs) or CD4+ cells, respectively. (A) Representative images of LCs (red, left panel) and CD4+ cells (green, right panel) shown. White bar = 10 μm. Cell nuclei stained with DAPI (blue). Only cells within the epithelium (above the basement membrane, denoted with white solid line and BM) were used in analysis. ES, dotted line, epithelial surface. (B) Probability density distributions using kernel density estimations of viral penetration depths from the epithelial surface after 4 hours (dotted red) and 24 hours (solid red) of exposure in inner (left) and outer (right) foreskins. Overlap of 24 hour penetrators and CD4+ cells (blue) in inner 2X greater than outer foreskin. (C) Cell count analysis shows greater numbers of CD4+ cells in inner (black squares) as compared to outer (white diamonds) foreskin (* p<0.05). (D) Analysis of cell depths show no difference between inner and outer foreskin. (E) Analysis of LCs in foreskin tissue before and after virus exposure in a subset of 4 donor samples. No difference seen in cell counts between inner and outer foreskin, but marginally more cells/image seen in inner foreskin after 24 hours of virus exposure (*p<0.05). (F) No difference in depths of cells before and after virus exposure, but this subset did have differences in LC depths between inner and outer foreskin at both time points. ***p<0.001

We also performed immunofluorescence imaging for tissue-resident immune cells by using foreskin tissues that had not been exposed to HIV-1 in culture and were immediately frozen upon arrival to the lab (Fig. 2A). We focused on cell phenotypes likely important in HIV sexual transmission: Langerhans cells (LCs) and CD4+ T-cell lymphocytes and macrophages[25–27]. Probability distributions of depths from the epithelial surface for both virions and cells were then used to estimate the likelihood that a penetrator would encounter an immune cell in the inner and outer foreskin. Given our finite dataset, we graphed normalized distributions using kernel density estimations (KDE) and then calculated the overlap of virus and cells in each tissue type (Fig. 2B). From this initial analysis, we found that the distribution of CD4+ cells in the tissue differed between the inner and outer foreskin, resulting in greater overlap of penetrating virions at 4 and 24 hours (S2 Fig.). In fact, there was a >2-fold greater overlap between penetrators and CD4+ cells in the inner as compared to the outer foreskin at 24 hours (S2G and S2H Fig.). We generally observed LCs abundantly in the epidermis, but no differences were seen in cell counts or depths between the inner and outer foreskin. To evaluate if these sentinel LCs might change in response to viral particles, we also analyzed their counts and depths after 24 hours of exposure to PA GFP HIV in a randomly selected subset of donors (n = 4). We found no difference in the overlap of virions and cells between the inner and outer foreskin in this subset at this time point (overlap percentages = 21.3 and 21.0, respectively, S2I Fig.).

Since the KDE distributions did not reflect varying virus stock concentrations used in each donor sample, number of obtainable images per sample, and repeated measures within samples, we developed models to make statistical comparisons between tissue types and time points. Our first model was constructed to evaluate total counts of virions per image, adjusted for virus stock concentration used for each tissue sample. Initial analysis took into account all images taken, including those in which no virions were seen (n = 1612 images). We found no difference between the inner and outer foreskin at 4 hours (mean 0.0505 +/− 0.0116 and 0.0542 +/− 0.0167 virions/image, respectively, Fig. 1E). This changed at the later time point, with more virions remaining in the inner as compared to outer foreskin (0.0495 +/− 0.0154 and 0.0171 +/− 0.0038 virions/image, *p* = 0.001). Correspondingly, a significant decrease in total virions from 4 to 24 hours was only seen in the outer foreskin (0.0542 +/− 0.0167 to 0.0171 +/− 0.0038 virions/image, *p*<0.001).

Our second model evaluated proportion of penetrators, adjusted for virus stock concentration. For this parameter, we evaluated only the subset of images in which at least one virion was seen, since no proportion could be calculated from an image where no virions were visualized (n = 964 images). We found no significant differences in the proportion of penetrators across tissue types or time points (Fig. 1F). We re-analyzed the first parameter with this subset of images and confirmed our findings from the initial analysis (i.e., more virions seen at 24 hours in the inner versus outer foreskin) (S1B Fig.). Our third model evaluated mean depths of penetration into the tissue and did not show any significant differences between the inner and outer foreskin (Fig. 1G). However, we observed significantly greater virion penetration depths at 24 hours as compared to 4 hours in both tissue types.

Using similar statistical models, we found more CD4+ cells in the inner as compared to the outer foreskin at baseline (mean 3.583 +/− 1.613 and 1.185 +/− 0.526 cells/image, respectively, *p* = 0.001), but no differences when comparing estimated mean depths between the two tissue types (Fig. 2C and 2D). There was no difference between the inner and outer foreskin in regards to total LCs or their depths from the epithelial surface at baseline (inner: 3.776+/−0.469 cells/image and 84.876 +/− 8.575 μm; outer: 4.060 +/− 0.689 cells/image and 89.240 +/− 11.869 μm, respectively). After 24 hours of virus exposure, we found a slight increase from baseline in the mean number of LCs in the inner foreskin (paired donors in the subset selected) (2.77 +/− 0.50 to 3.60 +/− 0.42 cells/image, p = 0.047, Fig. 2E) but the change in mean depths was not significant (89.70 +/− 3.26 to 74.31 +/− 9.63 μm, p = 0.129, Fig. 2F). In the outer foreskin, we found no significant changes in LC counts or depths after 24 hours of virus exposure. Comparing inner to outer foreskin, there was no significant difference in LC counts at either time point. Although outer foreskin LCs were closer to the surface as compared to those in the inner foreskin in this donor subset, the relative ratios did not significantly change after virus exposure (Fig. 2F).

### HIV-1 Interactions in Cadaveric Penile Tissues

**Fig 3:**
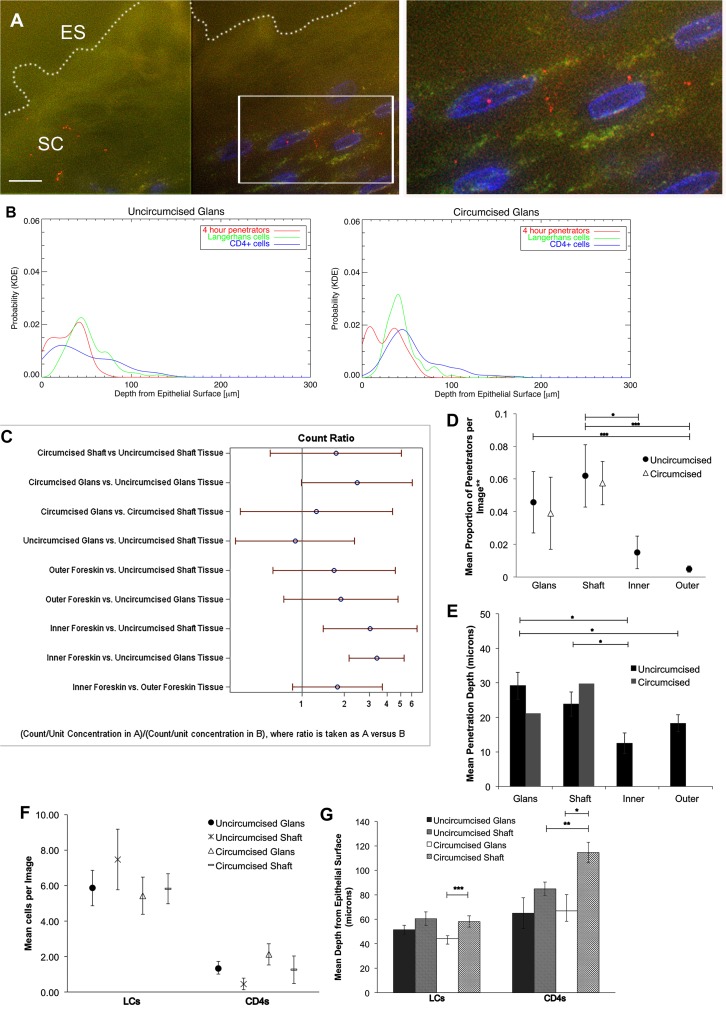
HIV-1 and immune cells in cadaveric penile epithelia. Penile tissues obtained from tissue donation organization banks inoculated with R5-tropic PA GFP-Vpr HIV-1 for 4 hours. (A) Representative image of glans tissue from uncircumcised donor after exposure in culture to HIV-1. Most virions were found on the epithelial surface (ES, white dotted line) in the SC. White bar = 10 μm. Cell nuclei stained with DAPI (blue). (B) Probability density distributions using kernel density estimations of viral penetration depths and tissue resident immune cells in uncircumcised glans (left) and circumcised glans (right). Overlap of 4 hour penetrators (red) and CD4+ cells (blue) appear different between tissues. (C) Interactions of estimated means of virions/image between tissue types and circumcision status, with log ratios presented for ease of reporting. Count ratios with CI >1 are considered statistically significant. (D) Estimated means of proportion of penetrators in tissues from uncircumcised (black circles) and circumcised donors (triangles). (E) Mean depth of virion penetration from uncircumcised (dark bars) and circumcised (gray bars) donors. Uncircumcised glans tissue allows higher proportion of penetrators than foreskin tissues and to greater depths. (F) Analysis of tissue resident immune cell counts shows more LCs found in epithelium than CD4+ cells. (G) Analysis of mean depths of cells shows LCs located more superficially in circumcised glans (white bar) versus shaft (gray dotted bar) and CD4+ cells more superficial in uncircumcised (gray hatched bar) as compared to circumcised shaft (gray dotted bar) tissues and in circumcised glans (white bar) versus shaft (gray dotted bar). *p<0.05, **p<0.01, ***p<0.001.

Beyond foreskin tissue, we sought to determine if differences existed between circumcised and uncircumcised penile tissues. We obtained 14 cadaveric penile specimens (7 uncircumcised and 7 circumcised) through tissue donation organizations. Tissue samples were cultured *ex vivo* with R5-tropic PA GFP HIV for 4 hours (we excluded longer incubation times due to potential tissue degradation from prolonged post-mortem tissue shipping) or immediately snap frozen as negative controls for immune cell analysis as described above. A total of 600 images were evaluated in the virion analysis (with 65% containing visible virions) and 352 images were used in the immune cell analysis. Similar to what was seen in the foreskin tissues described above, most visualized virions were on the surface, though penetrators could occasionally be seen between epithelial cells (average 3.4 per 100 virions) (Fig. 3A and S3 Fig.).

The same parameters described above were assessed in the penile explants. We observed differences in potential virus-cell interactions between uncircumcised and circumcised glans tissues using KDE plots of penetrators and immune cells (particularly with CD4+ cells) (Fig. 3B). However, calculated overlap percentages mainly showed differences between tissue and cell types, not circumcision status (S4 Fig.).

Using the statistical models described above, we compared estimated means of virions or cells between tissue types (glans, shaft, +/− inner and outer foreskin) or circumcision status. Again, because of potential tissue degradation after prolonged shipping times, we only included data from the 4 hour time point in this analysis. Data from the virion analysis are presented in Fig. 3C as ratios for ease of comparison across each variable (ratios >1 correlate with significant interactions). In the uncircumcised donor tissues, we found more virions/image in the inner foreskin than glans or shaft tissue (inner = 0.0507 +/− 0.0079 virions/image, glans = 0.0167 +/− 0.0033 virions/image, *p*<0.001, shaft = 0.0205 +/− 0.0065 *p* = 0.036). No difference was seen between inner and outer foreskins at this early time point, as was noted in the foreskin analysis from local donors. Re-analyzing the virion count with the subset of images that contained at least one virion confirmed this finding (n = 368 images, S3D Fig.). A larger proportion of penetrators was seen in the uncircumcised glans as compared to inner and outer foreskin (glans = 0.0458 +/− 0.0188 virions/image, inner = 0.0151 +/− 0.0100 virions/image, *p* = 0.099, and outer = 0.0048 +/− 0.0019 virions/image, *p*<0.001) (Fig. 3D). A significantly greater mean penetration depth was also seen in the uncircumcised glans tissue (29.162 +/− 3.908 μm), as compared to that in inner and outer foreskin tissues (12.466 +/− 2.985, p = 0.002 and 18.253 +/− 2.481 μm, p = 0.014, respectively). In this virion analysis, we observed no differences between the tissue types based on circumcision status for any of the parameters measured.

For the immune cell analysis, we found that uncircumcised glans epithelia contained marginally more CD4+ cells than shaft epithelia (1.333 +/ 0.387 vs. 0.452 +/− 0.323 cells/image, *p* = 0.05, Fig. 3F) and were closer to the epithelial surface though this was not statistically significant (64.892 +/− 12.584 vs. 84.883 +/− 5.587 μm, p = 0.158, Fig. 3G). We also observed CD4+ cells closer to the surface of shaft tissues from uncircumcised as compared to circumcised donors (84.883 +/− 5.587 vs. 114.500 +/− 8.437 μm, respectively, *p* = 0.003) and in the glans as compared to shaft tissue of circumcised donors (66.754 +/− 13.465 vs. 114.500 +/− 8.437 μm, respectively, *p* = 0.015). We did not observe significant differences in LCs counts between tissue types or circumcision status, but found that they were closest to the surface of the glans as compared to shaft tissue of circumcised donors (44.234 +/− 2.258 vs. 58.110 +/− 4.571 μm, respectively, *p*<0.001).

We also explored the urethral meatus (opening to the urethra, UM) as a potential site of HIV transmission. This area is continuous with the glans and is composed of non-keratinized stratified squamous epithelia[28]. We analyzed samples from 4 cadaveric donors (2 circumcised and 2 uncircumcised donors, but grouped them together as circumcision status should not affect this area) in which we could clearly delineate UM from the urethra and glans. The tissues were analyzed using the same methods as described above, except that we immunostained for CD68+ macrophages rather than LCs, as LCs are not found in the urethra. This subset included 48 images, with estimated means of 0.0319 +/− 0.0099 virions/image (adjusted for virus stock concentration, comparisons shown in S5B Fig.) and 0.0284 +/− 0.0229 penetrators/image; these values were not significantly different from those of other tissues analyzed. The mean penetration depth was significantly less than that observed in other tissues (7.583 +/− 1.729 μm, *p*≤0.001) except inner foreskin (p = 0.133), and the calculated overlap percentages from KDE plots of penetrators and immune cells was smaller than that observed in other tissues (S5C Fig.).

### HIV-1 in an *In Vivo* Rhesus Macaque Model

**Fig 4:**
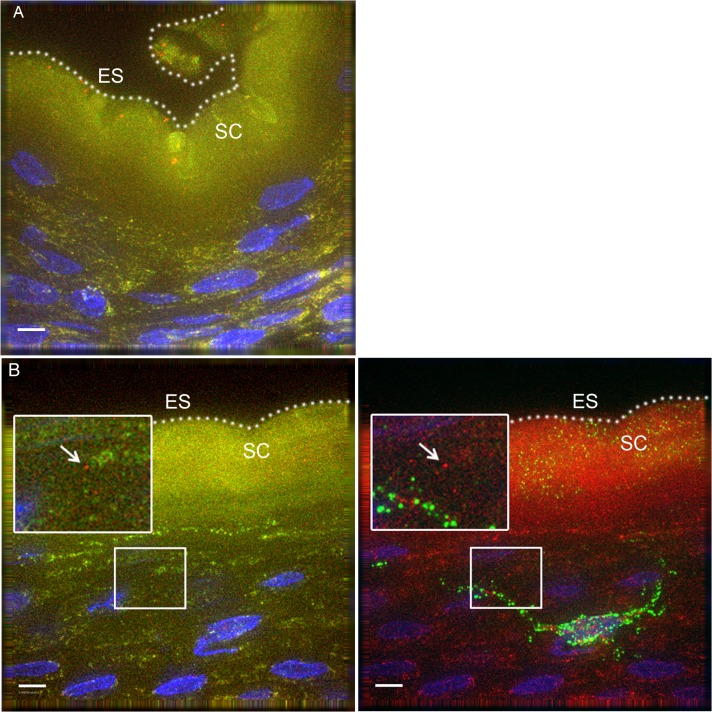
PA GFP HIV-1 interactions with rhesus macaque male penile tissues *in vivo*. Seven adult male rhesus macaques were inoculated with PA GFP HIV-1 *in vivo*. Penile tissues were obtained 4 hours after inoculation, cryosections immunostained for Langerhans cells, and imaged using epifluorescent microscopy. These experiments validated observations made with tissue explants using these techniques: similar to the *ex vivo* tissue culture model, most virions were seen attached to the epithelial surface (ES, dotted white line) or in the SC of the penile tissues, and relatively few virions were seen penetrating into the tissue. Representative images of (A) PA GFP HIV-1 in stratified squamous epithelium of macaque inner foreskin and (B) penetrating virion (red, left panel) near a superficial Langerhans cell (green, right panel) in inner foreskin tissue. Cell nuclei stained with DAPI (blue). White bar = 10 μm. Table shows summary data from images analyzed from all animals. Overall, significant inter-animal differences noted and hair follicles contributing to larger number of virions visualized in outer foreskin of these animals.

To determine if our observations may have been influenced by use of devitalized explant tissues, we sought an *in vivo* model to examine HIV interactions with intact penile epithelia[29]. To this end, we exposed 7 mature Indian male rhesus macaques (*macaca mulatta*) to PA GFP HIV using a “dunk” method as described in the methods section. Since these experiments were only intended to observe early interactions between virus and epithelium and to compare these observations to our *ex vivo* studies, we used the PA GFP HIV produced as described above. However, the animals were only exposed to viral supernatant for ∼15 minutes while anesthetized and allowed to resume normal activity for 4 hours prior to tissue collection. From these experiments, we obtained 1104 epifluorescent images of macaque penile tissues, which included 1552 individual visualized virions. We visualized PA GFP HIV interacting with macaque penile epithelia *in vivo* in a similar manner as with the *ex vivo* penile explant model (Fig. 4A and 4B). That is, the majority of viral particles remained on the surface or in the SC with a proportion able to penetrate into the epithelium. We used the statistical models described above to analyze the virions across tissue types and found a higher number of virions/image (0.01326 +/− 0.01247) but lower proportion of penetrators (0.02459 +/− 0.01015) in the outer foreskin as compared to other tissues (Table 1). Penetrators also reached greater depths in the outer foreskin (20.9262 +/− 7.1562 μm), significantly more so than in the glans tissues (*p =* 0.038). In the shaft tissues, we also observed high proportions of penetrators going to greater depths in the tissue (0.3803 +/− 0.1688 virions/image and 18.4040 +/− 6.2753 μm), but these observations may be attributed to specific macaque penile characteristics as described in the Discussion section below.

**Table 1:** PA GFP HIV-1 in macaque penile tissues *in vivo*.

	Virions/Image* +/− SE	Proportion of Penetrators/Image* +/− SE	Depth of Penetration into Epithelium (μm) +/− SE
**Inner Foreskin**	0.000585 +/− 0.000257	0.1038 +/− 0.05486	13.5462 +/− 3.4013
**Outer Foreskin**	0.01326 +/− 0.01247	0.02459 +/− 0.01015	20.9262 +/− 7.1562
**Glans**	0.000714 +/− 0.000305	0.2078 +/− 0.03256	4.7809 +/− 1.059
**Shaft**	0.00195 +/− 0.000106	0.3803 +/− 0.1688	18.404 +/− 6.2753

*Adjusted for virus stock concentration

## Discussion

While male circumcision has been shown to reduce HIV acquisition rates in men, we do not yet fully understand how this protection works, nor how the virus enters the male genital tract[3–5]. Plausible theories include the removal of a large surface area of tissue containing HIV-susceptible cells (the foreskin), but circumcised men still acquire HIV and it is unknown how penile transmission occurs after male circumcision[22]. To explore potential sites of HIV transmission across penile surfaces, we utilized epifluorescent microscopy to study PA GFP-labeled HIV-1 interactions with human tissue explants as well as in an *in vivo* rhesus macaque model[22,23]. In all penile tissues studied in both the human explant and macaque model, we observed most virions in the epithelial SC, even after 24 hours of exposure in culture. Co-inoculation of foreskin explants with HIV-1 and a fluorescent fluid phase marker (BSA) demonstrated that virions diffuse into the SC in a heterogenous pattern that is similar to the fluid phase marker. As no tissue washing occurred prior to fixation, we believe that this observation accurately reflects the simple diffusion of virions and BSA in culture. Furthermore, we observed similar diffusion patterns in the female macaque model upon exposure to BSA *in vivo[24]*.

In this study, we found significantly more HIV-1 viral particles remaining in the inner foreskin (predominantly in the SC) after 24 hours as compared to the outer foreskin. More viral particles were also seen within inner foreskin tissue as compared to other penile surfaces. We and others have demonstrated that inner foreskin SC thicknesses do not significantly differ from that of other foreskin areas and propose instead that a more physiological characteristic of the foreskin SC allows virions to perpetuate over time[13]. The persistence of virus in the inner foreskin may lead to infection in the uncircumcised male via two mechanisms: the first is that infectious viral particles are introduced into the urethral meatus after sexual intercourse, as the foreskin has been observed to cover the UM in the flaccid state in a proportion of uncircumcised men[30,31]. While our limited dataset of UM tissues did not indicate that this was a particularly vulnerable site, it is possible that larger datasets or analysis of other areas of the distal/anterior urethra may yield different results. (Although Ganor et al. have suggested that the “middle” urethra may be a site for HIV transmission, it is unclear how the virus would reach this area during or after sexual intercourse[32].) The second possibility is that retained viral particles enhance the immune response in the inner foreskin and adjacent glans (preputial space), which eventually leads to virion uptake by a superficial potential target cell in either tissue. The results of the Merck STEP study, where uncircumcised vaccine recipients exhibited the highest HIV acquisition rates, support such a dynamic preputial environment where immunologic changes in these tissues post-vaccination may have enhanced HIV transmission. Further supporting the existence of a dynamic preputial space are our observations that virions are able to penetrate the uncircumcised glans and inner foreskin epithelia to reach depths where LCs and CD4+ cells reside.

In fact, we demonstrated that penetrating virions could be seen reaching depths in several tissue types where resident immune cells were also found, particularly after 24 hours of culture. While it has been shown that LCs can be transiently infected by HIV-1 and transfer virions to CD4+ T-cells via synapses, we observed many penetrators at depths were CD4+ lymphocytes and macrophages could also be found[33,34]. The overlap of penetrators and CD4+ cells was greater in the inner as compared to outer foreskin, and in the uncircumcised glans as compared to other cadaveric penile tissues. Although we were unable to calculate the statistical significance of these distribution overlaps, they visually suggest that the inner foreskin and uncircumcised glans epithelia may be key sites in HIV transmission. These two surfaces form the preputial space in the uncircumcised man and the persistence of viral particles in the inner foreskin SC may lead to a greater likelihood of virion-target cell interactions within either tissue. We therefore propose that male circumcision protects against HIV transmission by not only removing the foreskin, but also by changing the remaining glans epithelium. Supporting this model is the observation that more virions were seen penetrating the uncircumcised glans epithelia and to greater depths than in the inner foreskin tissue. To our knowledge, this is the first study comparing circumcised and uncircumcised penile tissues, and future studies specifically evaluating glans epithelia using *in vivo* models or freshly obtained tissues will further investigate the role of this site in HIV transmission. Viral transmission across the glans epithelia might also explain how circumcised men remain at risk of HIV acquisition through the penis.

In deeper strata, dense intercellular junctions prevent the interstitial movement of foreign agents and accordingly, we observed only a small proportion of virions penetrating to these depths in all tissues evaluated[35]. The proportion of viral penetrators did not differ between the inner and outer foreskin from local donors at either early or late time points, nor in the cadaveric specimens at the only observed early time point. However, at the late time point, the absolute number of penetrators was higher in the inner foreskin of local donors given the greater total number of virions visualized there. Hypothetical mechanisms through which penetrators reached deeper epithelial strata include disruption of intercellular junctions, travel along or with LC processes, and/or epithelial cell trancytosis (though this has only been shown to occur through M cells in rectal epithelium)[33,36]. LC processes may also disrupt tight junctions themselves as they survey the external environment, and we have previously demonstrated that foreskin LCs can migrate in/out of foreskin epithelium in response to external agents[37,38]. However, we did not observe significant differences in LCs between inner and outer foreskin tissue, even after 24 hours of virus exposure, to explain the observed differences. We therefore hypothesize that at the early time point, most penetrators were quickly degraded by epithelial or immune cells and differences were only seen at the later time point after a saturation point between virions and cells had been achieved. With more virions persisting on the inner foreskin after 24 hours of culture, more would penetrate and be visibly intact in the tissue. Future studies evaluating live virus movement into fresh tissues will help to elucidate these potential mechanisms. Finally, the use of cadaveric specimens allowed us to uniquely compare tissues from circumcised and uncircumcised donors as well as between more penile sites, such as the UM. Due to the nature of our tissue collection process, we could not extend the cadaveric tissue cultures to the later time point as we did with freshly obtained foreskin tissues from local donors. However, the similarity in our observations between locally-obtained and cadaveric foreskins at the early time point suggest that longer term explant studies with freshly obtained penile tissues may uncover even greater differences between penile sites or donor circumcision statuses.

One caveat to using tissue explants is that observations may not reflect *in vivo* occurrences[29]. The rhesus macaque model, though somewhat different from humans, allowed us to verify that our observations were not an artifact of tissue explant cultures. With this model, we confirmed that virions can enter intact penile squamous epithelia, occasionally within reach of abundant LCs and CD4+ cells in the epidermis. As the macaque tissues were immediately snap-frozen in OCT, our observations likely reflect *in vivo* responses to virion exposure, rather than trauma from tissue excision. Of note, the data collected from the macaque experiments should be interpreted with caution due to key differences between macaque and human penile anatomy as well as experimental conditions. Firstly, the macaque outer foreskin is continuous with the abdominal skin and contains hair follicles, which traps viral particles. The foreskin also covers the entire length of the penis (starts at the proximal penis base), so the preputial space includes the shaft. This may lead to more virion accumulation and penetration in the macaque outer foreskin and shaft relative to other tissue types. Secondly, the animals were only exposed to viral supernatant while anesthetized. After this time and prior to necropsy, superficial virions were likely brushed off by the animal, resulting in fewer overall numbers of virions seen in the macaque model. Despite these differences, the use of the macaque model was important in verifying observations made in the tissue culture model. Furthermore, macaque models will be important in future studies examining infection of cells within the tissue, which require longer experimental times (days rather than hours) to achieve successful virus-tissue encounters and productive infection of the cell.

We also caution against directly comparing the results of this study to that previously published by our group utilizing the same virus identification technology to study the female reproductive tract of women and macaques[24]. Differences in methodology, such as the use of stitched panels in this study (as seen in S1 Fig.) and only counting penetrators seen past the SC (as many were seen within the SC) resulted in different counts and recorded depths of penetration. Our biostatisticians (AF and AR) also developed complex statistical models to include all images captured in the analysis (including many with zero counts) to make the comparisons reported, which was different from what had been done previously.

As noted in many other studies using donor tissues, we observed substantial heterogeneity between individuals in this study. For example, three specimens from three donors processed and inoculated on the same day with the same virus stock resulted in entirely different patterns of virus association and epithelial penetration. Factors contributing to this heterogeneity may include latent STIs such as HSV-2 or HPV, race, age, sexual activity or hygiene practices, which we did not collect information on in this study. Future studies examining these potentially confounding variables along with baseline skin structural/biological characteristics may help explain some of the observed inter-individual heterogeneity. Other drawbacks to our study include the use of tissues from men undergoing elective male circumcision or cadaveric donors. However, we took several measures to optimize the use of these specimens as described in the Materials & Methods section. We also saw no evidence of tissue degradation at the microscopic level in our image analysis.

In summary, we present data supporting that the inner foreskin may allow prolonged survival of infectious HIV particles in the preputial space, and that the uncircumcised glans penis may also be permissive to HIV encounters with CD4+ cells. This provides a mechanism for how male circumcision changes HIV susceptibility in a man, though further studies are needed to define how the glans tissue changes after male circumcision, as well as to demonstrate actual infection of immune cells within the tissue. Once a more complete model of HIV penile transmission is established, we may be able to devise other effective prevention strategies for HIV acquisition in men.

## Materials and Methods

### Ethics Statement

All work described in this study was reviewed and approved by the Institutional Review Board (IRB) and Animal Care and Use Committee of Northwestern University, the IRB of Rush Presbyterian Hospital, or by the ACUC at Tulane National Primate Research Center (TNPRC, protocol 0094). All human subjects provided written informed consent and all research was conducted according to the principles expressed in the Declaration of Helsinki. This study was carried out in strict accordance with the recommendations in the Guide for the Care and Use of Laboratory Animals of the National Institutes of Health (NIH) and with the recommendations of the Weatherall report; “The use of non-human primates in research." All procedures were performed under anesthesia using ketamine hydrochloride, and all efforts were made to minimize stress, improve housing conditions, and to provide enrichment opportunities (e.g., objects to manipulate in cage, varied food supplements, foraging and task-oriented feeding methods, interaction with caregivers and research staff). Animals were euthanized by ketamine hydrochloride injection followed by barbiturate overdose in accordance with the recommendations of the panel on Euthanasia of the American Veterinary Medical Association.

### Photoactivatable GFP-Vpr HIV-1

Green fluorescent protein linked to the N-terminus of HIV-1 Vpr was made as described by McDonald et al.[22]. To circumvent previous issues encountered with tissue auto-fluorescence, photoactivatable (PA) GFP, developed in the laboratories of Dr. Jennifer Lippincott-Schwartz, was incorporated to produce non-fluorescing GFP molecules that could be “turned on” by excitation and thereafter remain fluorescent[23]. Plasmids encoding PA GFP-Vpr and an HIV-1 provirus were used to co-transfect 293T cells and highly infectious viral supernatant was obtained at four hour intervals. Viral replication and infectivity was measured with HIV p24 ELISA assays and infectivity assays (mean 526.77 ng/ml p24). Viral stocks were stored at −80°C until ready for use.

### Foreskin Tissue Processing

Adult human foreskin tissues (n = 10 for 4 hour time point, n = 12 for 24 hour time point) were obtained from consenting adult donors. Donors were identified upon presentation for elective medical male circumcision through the Departments of Urology. We did not collect medical information such as presence of latent STIs on the subjects, though any subject with gross lesions were deferred for surgery. All tissue samples were de-identified prior to arrival to the laboratory. The de-identified tissue was processed within 2 hours of removal from the donor. Foreskin explants were washed with sterile 1X PBS (Hyclone), separated into inner and outer aspects, dissected into 0.5 x 0.5 x 0.2 cm sections, and placed individually into 24-well plates (Becton Dickinson). Sections were inoculated with 500 μl of PA GFP-Vpr HIV_Bal_ or HIV_R7_ supernatant and incubated at 37°C. At 4 and 24 hours, explants were removed from the supernatant, snap-frozen in OCT (Optimal Cutting Temperature, Sakura Finetek, Torrance, CA) compound in standard-sized plastic cryomolds (Sakura), and kept at −80°C for storage. Tissues were also snap-frozen in OCT and cryomolds without virus as negative controls.

### BSA

To evaluate tissue permeability and how virions might move into tissues, foreskin tissues were co-inoculated with PA GFP HIV-1 and fluorescently labeled bovine albumin serum (1 mg/ml, BSA, Sigma) at 37°C for 4 hours. BSA was labeled by direct conjugation to an amine reactive Alexa Fluor 594 dye (Molecular Probes, Invitrogen). Tissues were immediately snap-frozen in OCT after 4 hours of culture (with no washing prior to embedment) and stored at −80°C. Slides of cryosections were prepared as described below.

### Penile Tissue Processing

Cadaveric penile specimens (n = 14) were obtained from three tissue donation organizations: Life Legacy, ScienceCare, and National Disease Research Interchange (NDRI). Donors from these donation banks are screened at enrollment for pre-existing infections or medical conditions. To determine tissue viability, we inoculated tissue sections with 0.2% dinitrofluorobenzene (DNFB, Sigma-Aldrich) + RPMI + 10% fetal bovine serum for 4 hours at 37°C. Tissues were examined for CD1a cell expression, as this chemical is known to induce down-regulation of CD1a by LCs[38]. From these tests, we developed strict cut-offs of 36 hours post-mortem (donor time of death to tissue arrival in our Chicago laboratory) to ensure tissue viability for our *ex vivo* assays.

Specimens were washed with sterile 1X PBS, separated into glans, shaft, and if applicable, inner and outer foreskin, and further dissected into 0.5x0.5x0.2 cm pieces. Tissue pieces were placed into separate wells in plastic plates and inoculated as described above for foreskin specimens. Only 4 hour time points were evaluated in the analysis due to potentially significant tissue degradation at longer time points.

### Immunofluorescence

Thin (∼10μm) cryosections were placed onto 1mm glass slides (VWR) for immunostaining, kept frozen or immediately fixed with a PIPES-formaldehyde mix (0.1M PIPES buffer, pH 6.8 and 3.7% formaldehyde (Polysciences)) and washed with cold (4°C) 1X PBS. Tissues were blocked with 10% Normal Donkey Serum (NDS)/0.1% Triton X-100/0.01% NaN3. To examine virus location within the tissues, sections were immunofluorescently stained with Wheat Germ Agglutinin (WGA, Alexa Fluor 647, Invitrogen, 1μg/mL) and counterstained with 4,6-diamidino-2-phenylindole (DAPI, 1:25000).

LC immunofluorescent staining was performed with anti-human OKT6 (1:1) and anti-mouse donkey Rhodamine Red X (Jackson-ImmunoResearch, 1.5μg/mL). CD4+ immunostaining was performed with monoclonal anti-human mouse anti-CD4 antibody (Sigma, clone Q4120, 1:350 dilution) fluorescently conjugated with Alexa Fluor Zenon labeling kits (Invitrogen). CD68+ immunostaining was conducted with monoclonal anti-human mouse anti-CD68 antibody (Dako, clone EBM11, 1:200 dilution) and anti-mouse donkey Rhodamine Red X as above. Sections were counterstained with Hoescht as described above. Fluorescent mounting medium (DAKO, Denmark) and coverslips (VWR coverglass No. 1 thickness) were placed onto slides; slides were kept at 4°C until ready for imaging analysis.

### Epifluorescent Imaging and Analysis

All imaging was conducted with DeltaVision RT epifluorescent microscope systems (General Electric, Issaquah, WA). Virions were visualized with a 100x objective lens using either a 2x2 paneled fields of view (5.76e-2 mm^2^) to include the epithelial edge and as much of the epithelium as possible in the field of view, with roughly equal number of images obtained for each tissue type (at 4 hours, inner foreskin n = 357, outer foreskin n = 351; at 24 hours, inner foreskin n = 484, outer foreskin n = 420). For each donor sample, at least 10 images from three separate tissue sections taken ∼20 μm apart in the tissue block were obtained for analysis. Each area was surveyed three-dimensionally (30 z-stacks x 0.5 μm spacing). All images were deconvolved and analyzed with SoftwoRx software (GE) to identify virions and target cells in the tissue. PA GFP HIV-1 particles were identified using an inverse subtraction method: a pre-photoactivation image (accounting for background fluorescence) is captured and pseudo-colored as green, the field of interest is photoactivated with ∼495nm light, and a second post-photoactivation image is captured and pseudo-colored as red; the green and red images are overlaid and viral particles appear red whereas tissue auto-fluorescence appears yellow from the green and red overlap. Each viral particle identified was confirmed as such using the Line Profile feature on SoftwoRx, which allows individual measurements of fluorescence intensity at different wavelengths of light. Viral penetration into tissue was defined as visualization of a viral particle past the stratum corneum, since most virions were visualized in this layer. Measurements of viral penetration depth were taken using the two-point method, where a straight line was drawn between the viral particle and the closest point on the epithelial surface (SoftwoRx). Depths were calculated from a clear epithelial edge and only images in which an epithelial edge could be visualized were used in the analysis.

In the target cell analysis, slides of cryosections were prepared as described above and imaged using a 60X objective lens and epifluorescent microscopy. Cells were identified based on positive immunofluorescent signal, identification of a cell nucleus, and proper morphology. Cell counts were determined per image and area, and distances measured with the two-point method described above.

The probability distributions of penetrators and immune cells within the tissue were calculated using kernel density estimates of the respective probability distributions in each tissue type[39]. This was done without weighting for virus stock concentrations or total numbers of virions, cells or images. Graphs and overlap area calculations performed using Interactive Data Language (Exelisvus, CO, USA).

### Statistical Analysis

Our analysis of virions in tissue focused on three parameters: 1) total virion count per image, 2) proportion of penetrators per image, and 3) depth of penetration into the epithelium (from the epithelial surface) per penetrator. Separate models for each parameter were developed by biostatisticians (AF, FR) to best fit the observed data and account for repeated measures in each donor. We also accounted for virus stock concentration used with each donor sample—this was done with an offset option in the model statement for the total virion count and adjusted for the proportion of penetrating virions; therefore, the calculated estimated means reported for total virion count and proportion of penetrators are per unit of virus concentration (mean p24 = 526.77 ng/ml). For the first parameter, we used a Generalized Estimating Equation model (GEE) with a negative binomial distribution and logit link function for the foreskin tissue analysis and a GEE model with a zero-inflated negative binomial distribution and logit link function for the penile (including UM) analysis. Any virion seen on the epithelial surface, in the SC, or deep within the epithelium was included in this analysis. For the second parameter, we used a GEE model with a binomial distribution for the foreskin and penile analysis. This parameter was conditional for images in which at least one virion was visualized. To compare the first and second parameter, we analyzed the first parameter using the subset of images conditional on the presence of one virion. This was done with a zero-truncated negative binomial GEE model and the NLMixed function for both foreskin and penile datasets. We found no difference in the observations reported with the entire datasets for both the foreskin and penile data. The third parameter was analyzed using a GEE with a gamma distribution and log link model for the foreskin analysis and a GEE model with a gamma distribution for the penile analysis, conditional on images with at least one penetrator present. Each model was used to compare interactions between different tissue types, time points, and circumcision statuses.

The target cell analysis was conducted with similar models. Specifically, foreskin and penile cell counts were analyzed with a GEE model with a negative binomial distribution and cell distances from the epithelial surface were analyzed with a GEE model with a gamma distribution. Each model was used to compare interactions between tissue types and circumcised statuses. All analysis was conducted with SAS 9.3 with an alpha of 0.05.

### Rhesus Macaque Model

The animals used in this study were housed at the TNPRC in Covington, LA in accordance with the regulations of the American Association for Accreditation of Laboratory Animal Care. Animals were anesthetized as described above and inoculated with ∼1.5mls of PA GFP HIV-1 supernatant (from the same stocks as described above in the tissue culture explant model) for at least 15 minutes. Virus inoculations were performed by manually retracting the foreskin then submerging the penis in viral supernatant for the duration of the inoculation. The animals were then taken off sedation and allowed to resume normal activity for 4 hours before euthanization and necropsies were performed. Penile tissues were separated into glans, shaft, inner and outer foreskin and smaller sections individually snap frozen in plastic cryomolds containing OCT. Frozen tissue blocks were then shipped to Northwestern and cryosections obtained as described above. Images were acquired and analyzed using DeltaVision RT systems and SoftwoRx software as described above.

## Supporting Information

S1 Fig PA HIV-1 in foreskin tissue. (A) In occasional images, penetrating virions were found deep in inner foreskin epithelia, almost reaching the basement membrane (BM). ES, epithelial surface, dotted white line. SC, stratum corneum. White bar = 10 μm, blue = cell nuclei. (B) Analysis of virion counts (** = adjusted for virus stock concentration) using the subset of images with at least one penetrator in order to compare to analysis of proportion of penetrators showed similar results as analysis with total dataset. *p<0.05, **p<0.01.
(TIF)

S2 Fig Probability distributions of virions and immune cells in foreskin tissue from local donors. (A-H) Probability density distributions using kernel density estimations of viral penetration depths (red) at 4 (A, B, E, F) and 24 hours (C, D, G, H) and tissue resident immune cells, CD4+ cells and LCs (green) at baseline (no virus exposure). Percentage of overlap between areas of penetrators and cells reported in blue. (I) Overlap of penetrators and LCs after 24 hours of virus exposure in a subset of foreskin donors (n = 4). Highest overlap seen between 24 hour penetrators and CD4+ cells in inner foreskin (C). Lowest seen between 4 hour penetrators and CD4+ cells in outer foreskin (B).
(TIF)

S3 Fig PA HIV-1 in cadaveric penile tissues. (A-C) Representative images of uncircumcised shaft (A), circumcised glans (B), and circumcised shaft tissues (C), respectively. ES, epithelial surface, dotted white line. SC, stratum corneum. White bars = 10 μm. Cell nuclei stained with DAPI (blue). (D) Analysis of virion counts (** = adjusted for virus stock concentration) using the subset of images with at least one penetrator in order to compare to analysis of proportion of penetrators showed similar results as analysis with total dataset. *p<0.05, ***p<0.001.
(TIF)

S4 Fig Probability distributions of virions and immune cells in cadaveric penile tissues. Probability density distributions using kernel density estimations of viral penetration depths (red) and tissue resident immune cells (green). Percentage of overlap / area of virion curve reported in blue. Highest overlap seen between 4 hour penetrators and LCs in uncircumcised glans (top left). Lowest seen between 4 hour penetrators and CD4+ cells in circumcised shaft (bottom right).
(TIF)

S5 Fig Virions and immune cells in urethral meatus. (A) Representative image of PA HIV-1 (red) in/on urethral meatal (UM) tissue from circumcised donor. Most virions were also found on the epithelial surface (ES, dotted white line) of this non-keratinized stratified squamous epithelium (white arrows point to two virions). Immune cells (green, CD4+) were found closer to the basement membrane (BM, solid white line). White bar = 10 μm. Cell nuclei = blue. (B) Interactions of estimated means of virions/image (adjusted for virus stock concentration) between UM and other tissue types, with log ratios presented for ease of reporting. (C) Probability density distributions using KDEs of viral penetration depths (red) and tissue resident immune cells (green, CD4+ in top graph, CD68+ in bottom graph) in UM tissue. Overlap percentages (blue) were less than that seen in other tissue types.
(TIF)
