# Supplementary figures and images for: Visualization of HIV-1 Interactions with Penile and Foreskin Epithelia: Clues for Female-to-Male HIV Transmission
Source: PLoS Pathog. 2015 Mar 6;11(3):e1004729. doi: 10.1371/journal.ppat.1004729 (PMC4352059; doi:10.1371/journal.ppat.1004729)

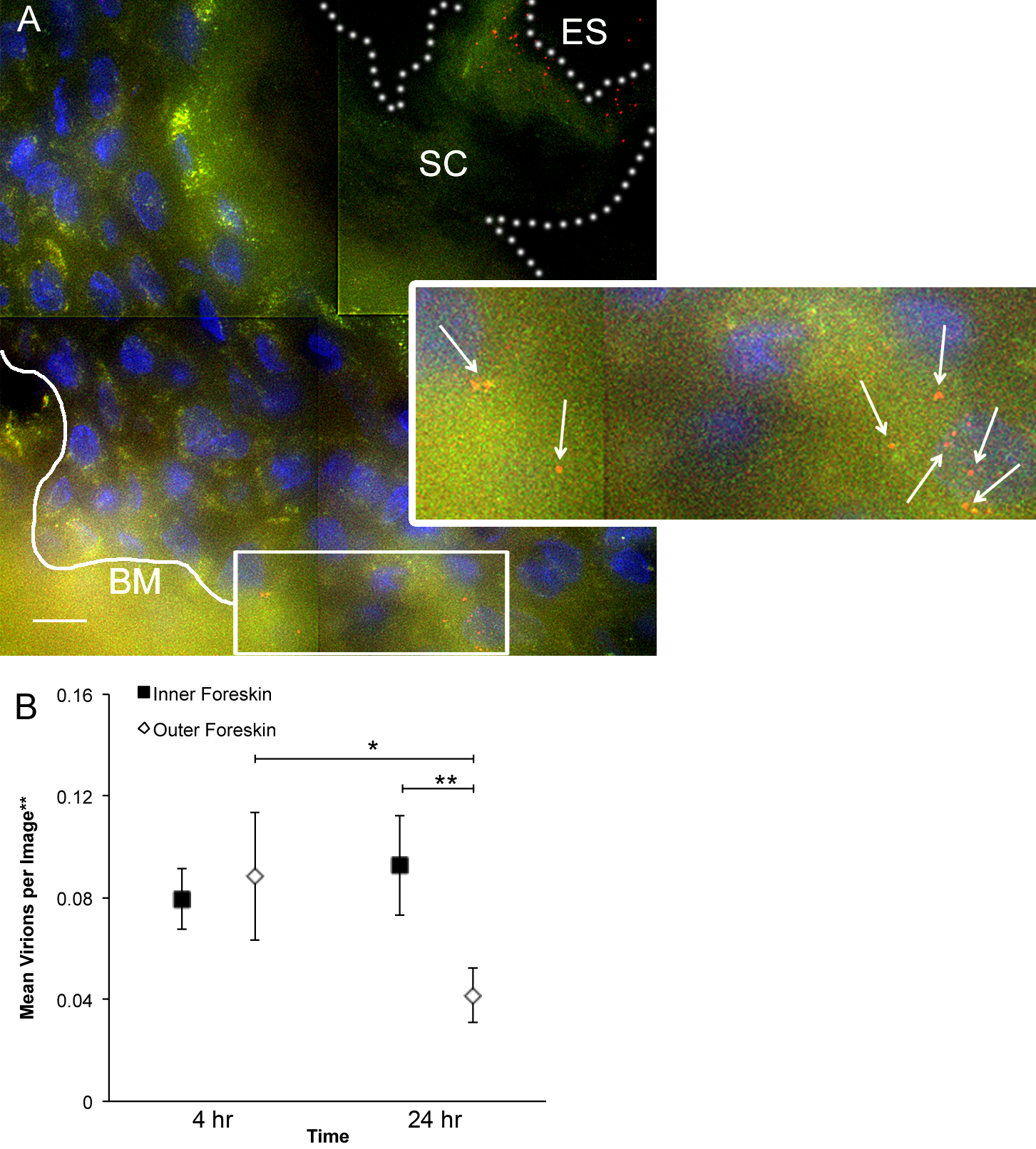

Supplement: S1 Fig — (A) In occasional images, penetrating virions were found deep in inner foreskin epithelia, almost reaching the basement membrane (BM). ES, epithelial surface, dotted white line. SC, stratum corneum. White bar = 10 μm, blue = cell nuclei. (B) Analysis of virion counts (** = adjusted for virus stock concentration) using the subset of images with at least one penetrator in order to compare to analysis of proportion of penetrators showed similar results as analysis with total dataset. *p<0.05, **p<0.01. (TIF) [file ppat.1004729.s001.tif]

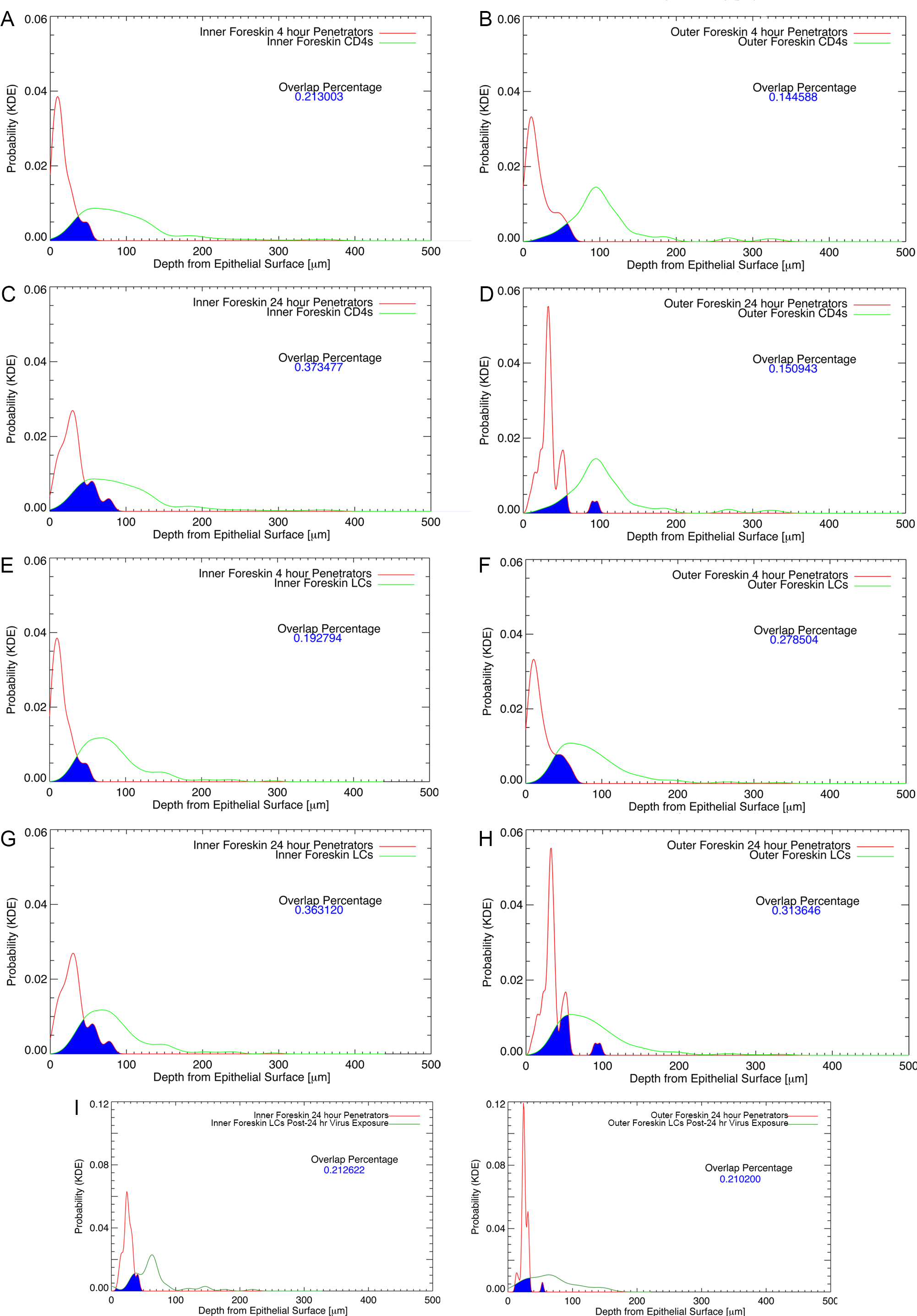

Supplement: S2 Fig — (A-H) Probability density distributions using kernel density estimations of viral penetration depths (red) at 4 (A, B, E, F) and 24 hours (C, D, G, H) and tissue resident immune cells, CD4+ cells and LCs (green) at baseline (no virus exposure). Percentage of overlap between areas of penetrators and cells reported in blue. (I) Overlap of penetrators and LCs after 24 hours of virus exposure in a subset of foreskin donors (n = 4). Highest overlap seen between 24 hour penetrators and CD4+ cells in inner foreskin (C). Lowest seen between 4 hour penetrators and CD4+ cells in outer foreskin (B). (TIF) [file ppat.1004729.s002.tif]

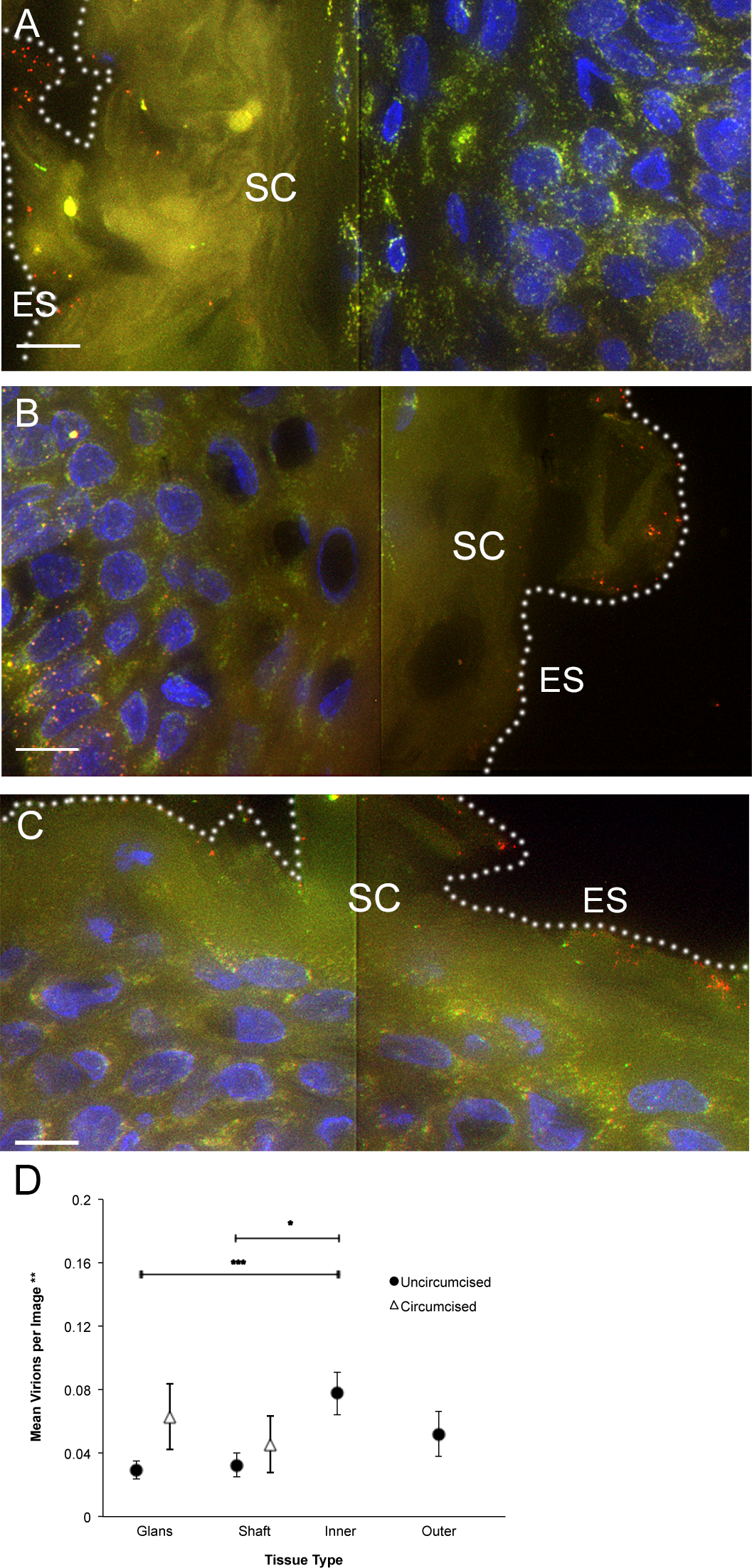

Supplement: S3 Fig — (A-C) Representative images of uncircumcised shaft (A), circumcised glans (B), and circumcised shaft tissues (C), respectively. ES, epithelial surface, dotted white line. SC, stratum corneum. White bars = 10 μm. Cell nuclei stained with DAPI (blue). (D) Analysis of virion counts (** = adjusted for virus stock concentration) using the subset of images with at least one penetrator in order to compare to analysis of proportion of penetrators showed similar results as analysis with total dataset. *p<0.05, ***p<0.001. (TIF) [file ppat.1004729.s003.tif]

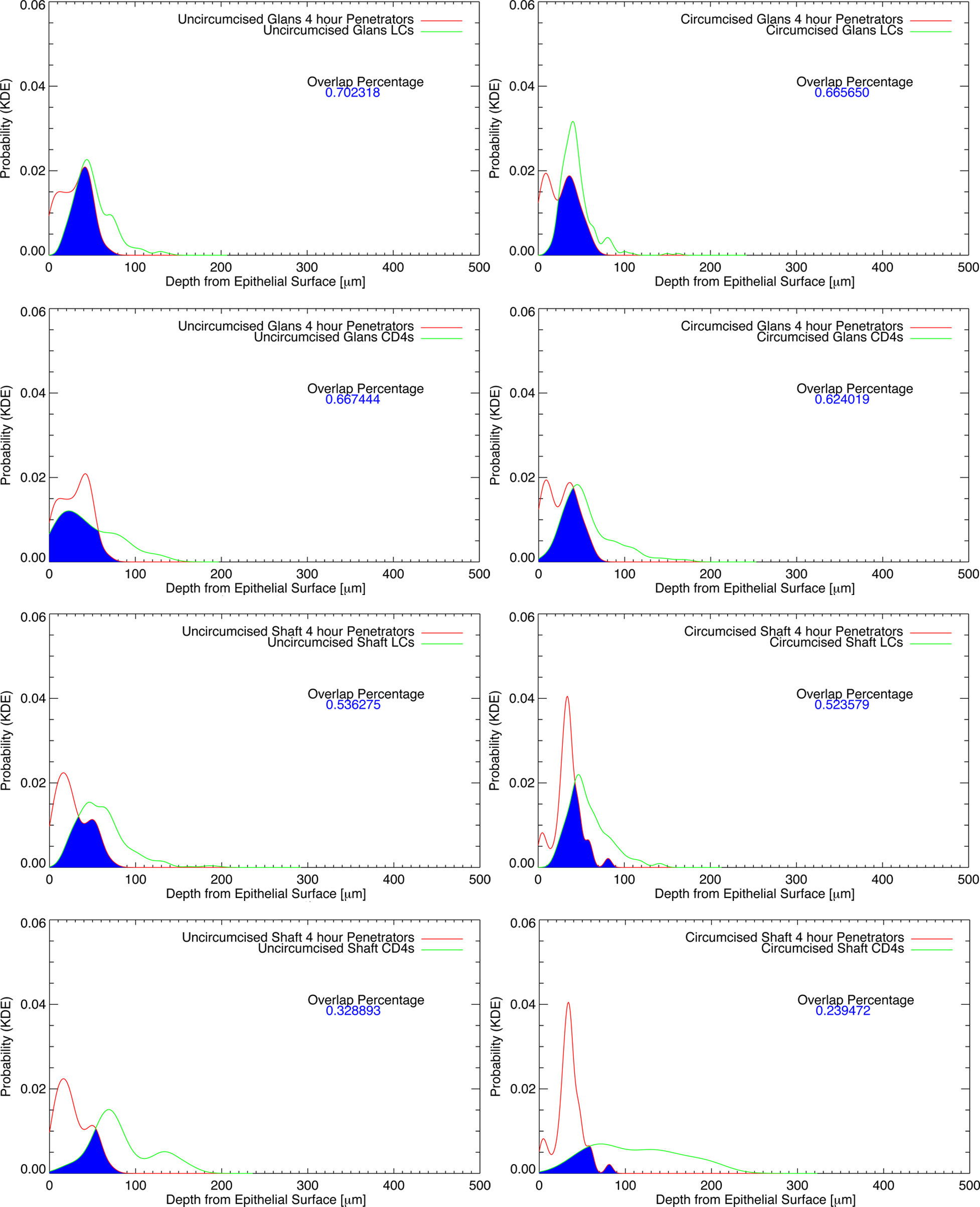

Supplement: S4 Fig — Probability density distributions using kernel density estimations of viral penetration depths (red) and tissue resident immune cells (green). Percentage of overlap / area of virion curve reported in blue. Highest overlap seen between 4 hour penetrators and LCs in uncircumcised glans (top left). Lowest seen between 4 hour penetrators and CD4+ cells in circumcised shaft (bottom right). (TIF) [file ppat.1004729.s004.tif]

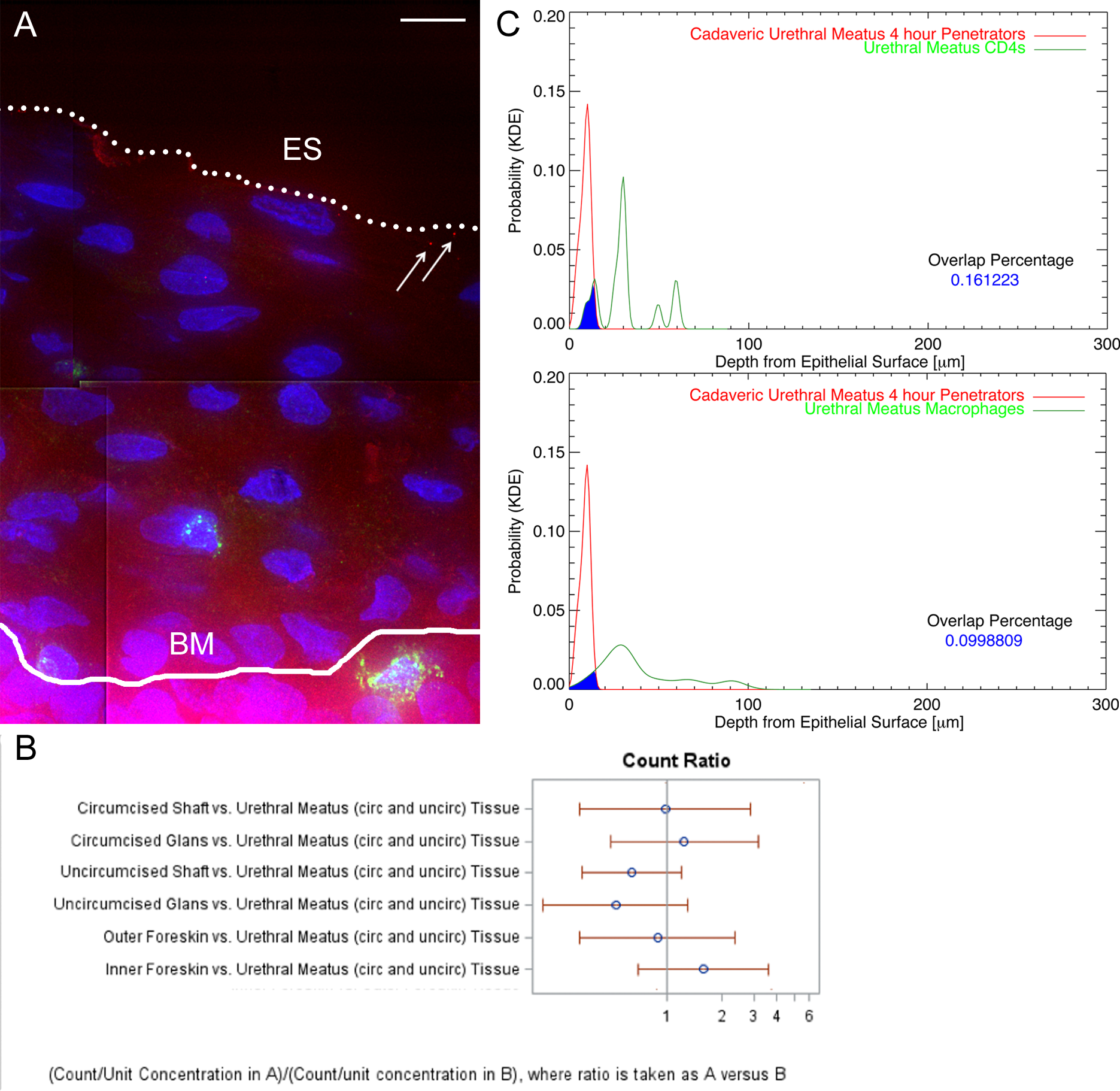

Supplement: S5 Fig — (A) Representative image of PA HIV-1 (red) in/on urethral meatal (UM) tissue from circumcised donor. Most virions were also found on the epithelial surface (ES, dotted white line) of this non-keratinized stratified squamous epithelium (white arrows point to two virions). Immune cells (green, CD4+) were found closer to the basement membrane (BM, solid white line). White bar = 10 μm. Cell nuclei = blue. (B) Interactions of estimated means of virions/image (adjusted for virus stock concentration) between UM and other tissue types, with log ratios presented for ease of reporting. (C) Probability density distributions using KDEs of viral penetration depths (red) and tissue resident immune cells (green, CD4+ in top graph, CD68+ in bottom graph) in UM tissue. Overlap percentages (blue) were less than that seen in other tissue types. (TIF) [file ppat.1004729.s005.tif]
